# Assessment of concentrated solar power (CSP) generation potential in Cameroon using a Multi-Criteria Analysis Method and Geographic Information System (GIS)

**DOI:** 10.1016/j.heliyon.2024.e41094

**Published:** 2024-12-13

**Authors:** Fotsing Metegam Isabelle Flora

**Affiliations:** a Environmental Energy Technologies Laboratory (EETL), Department of Physics, University of Yaounde I, P.O Box 812, Yaounde, Cameroon; b Department of Energetic, Environment and Thermal Engineering, UR-ISIE, University Institute of Technology Fotso Victor, University of Dschang, P.O Box 134, Bandjoun, Cameroon

**Keywords:** Solar CSP, MCDM-AHP, GIS, Cameroon, Sensitive analysis

## Abstract

This research aims to identify wet-cooled CSP (Concentrated Solar Power) solar power plants connected to the existing electricity grid in Cameroon. This study uses a hybrid approach which combines an MDCM-AHP method (Multi-Criteria Analysis Method – Hierarchical Analysis Process) and a GIS (Geographic Information System). The elements studied are the climate (Direct Normal Irradiance (DNI), temperature), orography (slope and elevation) and location (proximity to the electricity network, proximity to roads and railways, proximity to homes), in order to determine the weight of these different factors and combine them to obtain the final map. The method was used to map and visualize unsuitable areas, as well as to rank suitable sites. According to the results, over 42.35 % of the country's nland is unsuitable for the construction of CSP solar plants, mainly due to excessive land use. According to the results, 0,001 % of Cameroon's land is considered “Less suitable”, 44 % “Suitable”, 13.46 % “Highly suitable” and 0.01 % “Most suitable".The sensitivity study includes three different situations, namely the technical scenario (high consideration of technical factors such as DNI, temperature, elevation, and slope). The economic scenario (where economic factors such as proximity to electricity grids, roads, residential areas and watercourses are taken into account) and the equal-weight scenario (where all factors are equal) clearly demonstrate that we can find very suitable areas for the installation of CSP solar plants in Cameroon, particularly in the Far North region. A detailed analysis by region shows that the theoretical potential for CSP solar energy in regions with “Less Suitable”, “Suitable”, “Highly Suitable” and “Most Suitable” suitability is approximately 4242.25 TWh/year, 29328.658 TWh/year, 3965.25 TWh/year and 5.794 TWh/year. The overall potential amounts to approximately 37536.159 TWh/year for all ten Canadian regions. Thanks to the model, it has been possible to identify the most suitable regions for CSP investment and offers opportunities to carry out more in-depth studies in order to choose the right site.

## Highlights

• The issues identifying optimal sites for solar CSP-based smart green electricity production in Cameroon.
• The proposed framework employs a hybrid MCDM-AHP and GIS approach to identify suitable locations for wet-cooled CSP plants, considering climate, topography, and infrastructure.
• The study reveals that 57.65 % of the areas are suitable for solar CSP power plant construction.
• The estimated CSP energy potential across regions is 37,536.16 TWh/year, identifying key areas for investment.
• Three scenarios highlight suitable areas for CSP development, particularly in Cameroon's Far North region.

## 1 Introduction

Over the past decade, numerous solar projects have been initiated across Africa, showcasing the market's robustness amidst the COVID-19 pandemic. A 2021 PwC report indicates that only 58 % of Africans have access to electricity [1]. To satisfy the continent's demands, Africa is expected to double its energy production by 2030 and increase it fivefold by 2050. As far as the energy transition is concerned, this should focus mainly on renewable energies, with a predicted 110 % increase in solar energy by 2050, due in particular to a very high annual sunshine rate [1]. In 2022, solar projects will continue to be developed in Benin, Burkina Faso, Cameroon, Chad, Ivory Coast, Mali, Senegal, and North Africa, but the resource is still underutilized, with most solar installations not exceeding 50 MW, which is too low to meet the energy needs of major cities. One possible solution is to develop large-scale national production infrastructures, but this requires identifying areas with high technical and economic potential and, above all, improving the distribution network. Today, more than a billion people are deprived of electricity, more than half of whom live in sub-Saharan Africa. This is due to the lack of information about renewable energy in Africa and the absence of in-depth research and scientific investigation into the integration of renewable energy technologies [2].

Climate change and the rising costs of fossil fuel use have led to the integration of solar photovoltaic and wind power into the global energy mix, but the intermittency experienced when integrating these two types of energy into existing electricity grids has highlighted several obstacles. The construction of solar or wind power plants requires the use of storage resources, which account for around 40 % of the cost of the installation. Energy storage plays an essential role in the success of any intermittent energy source, whether solar or wind. Concentrated solar power (CSP) is the only renewable energy source that allows storage without batteries [3]. The CSP solar plant generates electricity using thermal heat, and the heat source is direct normal irradiance (DNI). Integrating heat storage into the CSP makes it possible to generate electricity during hours when there is little or no solar energy. It should be emphasized that the choice of heating plays a key role in the efficiency and smooth operation of a CSP plant. CSP plants mainly use two cooling methods: wet recirculation cooling and dry cooling [4]. Dry cooling uses air rather than water for cooling and requires a minimum level of water for cleaning the condenser tube bundles. Its main disadvantage is the higher price of electricity. Indeed, the use of dry cooling in CSP plants leads to a reduction in the efficiency of these power plants and increased capital expenditure. Depending on location, this type of cooling can lead to an increase in production costs ranging from 7.87 % to 5.65 % for hot and cool climates [4]. The wet cooling method is particularly effective as it allows the heat from the steam to be returned to the air through the evaporation of the cooling water. This type of cooling is less affected by fluctuations in ambient air temperature than dry cooling, but it requires almost 10 times more water than dry cooling and needs to be built close to high-flow water sources. This study will utilize wet recirculation cooling because it is the predominant method (employed by 80 % of operational facilities), it provides superior efficiency, and it entails lower investment costs than dry cooling [4].

Despite Cameroon's vast potential for renewable resources, particularly solar PV, the nation still relies heavily on fossil fuels for electricity production in regions beyond the existing power grid and for backup power plants. However, in the past decade, the Cameroonian government has shown interest in devising a strategy for energy diversification that leverages renewable technologies and has been proactive in advocating for its renewable resources. This has resulted in the implementation of favorable policies to foster the growth of renewable energy, thereby mitigating environmental issues associated with greenhouse gas (GHG) emissions. Consequently, the government has embraced the Electricity Sector Development Plan for 2030 (PDSE 2030), initiated in 2011, which aims to diversify energy sources and boost economic expansion. The PDSE 2030 plan places a strong emphasis on renewable energies, especially solar power, to fulfill its goals. Through the Regional Off-Grid Electricity Access Project (Rogeap), funded by the World Bank, the government provides subsidies to entrepreneurs and SMEs in the renewable energy sector, with amounts up to 153 million FCFA [5]. While Cameroon has a high reliance on hydroelectric power generation, unconventional renewable energy sources such as solar and wind power have yet to make a significant contribution to the nation's power systems [6]⁠. About 80 % of the energy in Cameroon comes from hydroelectric power, 14 % from gas, and 6 % from solar, wind, and biomass energy [5]. Before promoting the use of renewable energy in Cameroon, it will be necessary to assess each energy source's potential. This can be achieved by employing spatial tools such as Geographic Information Systems (GIS) [7]. Due to the uneven spatial distribution of renewable energy sources, their full potential can only be harnessed through proper utilization [8]⁠. As the global expansion of renewable energy projects accelerates, it becomes crucial to select sites that are favorable for their deployment, focusing on reducing installation costs and minimizing the environmental impact of their development [9]. Concentrated Solar Power (CSP) sources, capable of producing and storing thermal energy from unrestricted solar radiation, are regarded as a viable alternative to fossil fuels due to their environmental benefits and low greenhouse gas emissions. However, a major limitation is their unavailability in every region. The identification of suitable areas for CSP solar energy systems is a decision-making challenge that requires evaluating the resource's potential alongside technical, economic, and environmental considerations [10].

Decision-making based on multiple criteria analysis (MCDM) is a set of techniques that helps decision-makers make complex decisions and weigh their possibilities. Geographical problems are frequently analyzed thanks to techniques based on geographic information systems (GIS) [11]⁠. Various studies have used multi-criteria analysis approaches connected to GIS to represent areas that are conducive to the building of CSP solar parks in various parts of the world [2], [6], (12–19). According to Table 1, some extensive studies have combined MCDM-GIS in various countries and regions to determine the best location for installing solar PV and CSP power plants. According to these studies, it is clear that selecting ideal areas for the installation of CSP solar power plants is not an easy task, given the number and variety of criteria that are considered in this decision. The decision criteria used in previous studies on CSP solar plants are shown in Table 2. Table 2 shows that the most common criteria for identifying large-scale CSP solar parks connected to the existing electricity network are Direct Normal Irradiation (DNI), proximity to the electricity network, proximity to roads, proximity to residential areas, proximity to watercourses, and slope.

**Table 1:** Studies using combined GIS and decision making for solar PV and CSP site selection.

	Authors	Year	Solar-power technologies	Criteria	Case study	Methods
1	Georgiou and Skarlatos [12]⁠	2016	PV	10	Cyprus	GIS and AHP
2	Al Garni et al. [13]⁠	2017	PV	7	Saudi	GIS and
					Arabia	AHP
3	Aly et al. [2]⁠	2017	PV and CSP	6	Tanzania	GIS and
						MCDM
4	Doljak and Stanojević [14]⁠	2017	PV	7	Serbia	GIS and
						AHP
5	Liu et al. [15]⁠	2017	PV	8	China	Grey cumulative
6	Zoghi et al. [16]⁠	2017	PV	15	Iran	GIS and AHP
7	Suuronen et al. [17]⁠	2017	PV	12	Chile	GIS and AHP
8	Wang et al. [18]⁠	2018	PV	15	Vietnam	Fuzzy AHP, DEA, and TOPSIS
9	Ozdemir and Sahin [19]⁠	2018	PV	5	Turkey	GIS and AHP
11	Fang et al. [20]⁠	2018	PV	10	China	Rough PT-based TOPSIS
12	Merrouni et al. [21]⁠	2017	PV	8	Morocco	GIS and AHP
13	Yousefi et al. [22]	2018	PV	9	Iran	GIS and Boolean-Fuzzy
14	Alisa Yushchenko et al. [23]⁠	2018	PV and CSP	5	West Africa	GIS and AHP
15	Doorga et al. [24]⁠	2018	PV	9	Mauritius	GIS and MCDM, sensitivity analysis
16	Majumdar and Pasqualetti [25]⁠	2019	PV	9	USA	GIS and Multi-Criteria Analysis
17	Marina Giamalaki et al. [26]⁠	2019	PV and CSP	10	Mediterranean	GIS and AHP, sensitivity analysis
18	Solangi et al. [27]⁠	2019	PV	20	Pakistan	AHP and Fuzzy VIKOR
19	Ghasemi et al.	2019	PV and CSP	9	Iran	GIS and MCDM
20	Giamalaki and Tsoutsos	2019	PV and CSP	9	Greece	GIS and AHP
21	Colak et al. [28]⁠	2020	PV	10	Turkey	GIS and AHP
22	Lijian Sun et al. [29]⁠	2020	CSP	7	China	GIS and MCDM-AHP
23	Fotsing Isabelle et al. [6]⁠⁠	2020	PV and CSP	12	Cameroon	GIS-Boolean
24	Haddad et al. [27]⁠	2021	CSP	7	Algeria	GIS and AHP
25	Lindberg et al. [30]⁠	2021	PV	/	Sweden	GIS
26	Soydan	2021	PV	11	Turkey	GIS and AHP
27	O. Lindberg et al. [30]⁠	2021	PV	6	Swedish municipality	GIS- power flow analysis
28	Foad Minaei et al. [31]⁠	2021	PV	10	Iran (Khorasan-e-Razavi)	GIS-fuzzy best worst
29	Gouareh et al. [32]⁠	2021	CSP	/	Algeria	GIS and AHP and best worst

**Table 2:** Decision criteria considered in the previous studies solar CSP plants.

Criteria	[2]⁠	⁠ [29]⁠	[33]⁠	[34]⁠	[35]⁠	[36]⁠	[2]⁠	[37]⁠	[32]⁠	[6]⁠⁠	[38]⁠
Solar resources DNI	×	×	×	×	×	×	×	×	×	×	×
Slope		×		×	×	×		×		×	×
Aspect				×							
Elevation									×	×	
Temperature										×	
Proximity to water bodies	×	×	×	×	×	×	×		×	×	×
Proximity to airports					×					×	×
Proximity to wildlife designations	×	×	×	×	×	×	×		×		×
land-use	×	×	×	×	×	×	×		×	×	×
Proximity to residential area	×	×	×	×	×	×	×		×	×	×
Proximity to roads	×	×	×	×	×	×	×		×	×	×
Proximity to transmission lines	×	×	×	×	×	×	×		×	×	×
Population density			×							×	×
Farm required area					×					×	

Despite their importance in the selection of ideal sites for large-scale solar CSP, previous studies have rarely included population density and the required surface area. According to Table 2, the AHP analytical hierarchy method is one of the most commonly used methods for this type of research. According to Ref. [39]⁠ [40]⁠ [41], AHP is the most commonly used method to classify PV and CSP solar site choices. Many authors have used MCDM coupled with GIS to identify large-scale CSP parks connected to the existing electrical network.

For instance, L. Sun, Y. Jiang et al., used the AHP-GIS method to evaluate the potential and select suitable locations for solar PV and CSP parks in Ningxia, China, by taking into account seven criteria: solar irradiation DNI, average temperature, slope, proximity to a river, proximity to roads and railroads, proximity to the power grid, and proximity to a load demand area. The results show that the combined technical potential of the PV and CSP systems is estimated to be 443 TWh/year and 308 TWh/year, respectively. They claim that, despite the technology's significant technical potential in CSP, its limited water resources make it less suitable for the study area than photovoltaic technology [29]. The warm CSP and PV points in Tanzania have been identified by A. Aly et al., thanks to the AHP-GIS method, which is based on seven criteria: average temperature, slope, solar irradiation DNI, closeness to a river, closeness to a road or railroad, closeness to a power grid, and closeness to a load demand area. Based on the findings, four hot spots are seen for CSP installations and four hot spots for photovoltaic installations [2].A. Yushchenko et al., presents the geographical and technical estimates of solar electricity production potential connected to the electrical and hybrid networks in Western Africa. This study combined the MCDM and GIS methods by focusing on five factors: population density, solar irradiance (DNI), proximity to roads, communities, and the power grid. They claim that the findings may be used to represent potential interest areas for the expansion of the electrical grid and the implementation of solar energy production [23]. K. Tlhalerwa and M. Mulalu, evaluated the technical potential of solar energy in Botswana using an ascending approach that takes into account resource constraints and the availability of solar energy. Land exclusion criteria based on extensive geographic information systems (GIS) and land use data were used to assess the potential of CSP in the ten districts of the country. The findings show that there is roughly 220,016 km^2^ of land available in Botswana for the construction of CSP centers, with a nominal capacity estimated at 363,86 GW [42]. A. Gouareh, B. Settou, and N. Settou, developed an alternate framework in Algeria for selecting large-scale CSP centers' locations that concentrate solar energy on the network using the MCDM-GIS method based on seven criteria (solar irradiation DNI, land use, slope, sunshine duration, proximity to roads, proximity to the power grid, proximity to urban areas). The sensitivity analysis is conducted using three scenario tests that consider the diverse perspectives of various energy-related groups. These tests examine the sensitivity by accounting for the different viewpoints on energy. Several evaluation techniques are employed, including the Best Worst Method (BWM), the Analytic Hierarchy Process (AHP), and Equal Weighting (EQW). The results of the study suggest that approximately 11 % of the study area is well-suited for the installation of large-scale Concentrated Solar Power (CSP) parks connected to the electrical grid, with an estimated annual electricity generation of about 34,453 TWh [32]. B. Haddad et al., still in Algeria, proposed a hybrid method to identify suitable locations for building solar CSP centers by combining multicriteria AHP decision-making with geographic information systems (GIS). The study's findings indicate that about 51 % of the country's land is unsuitable for the establishment of CSP solar parks. The seven criteria used in this study are sun irradiation (DNI); slope orientation; slope; closeness to a river; closeness to a road or railroad; closeness to a power grid; and closeness to a high-density area [38]. Y. Charabi and A. Gastli, primarily used GIS to create the solar map of a significant Oman plant in Duqum, based on topographic and geographic data from the area. The results show that the estimated annual potential electricity generation is roughly 2,3 TWh [37]. Additionally, A. Alami et al., employed the AHP-GIS method to assess potential and select CSP locations in the eastern part of Morocco based on eight criteria: solar irradiation (DNI), proximity to dams, slope, proximity to waterways, proximity to roads and railways, proximity to the power grid, proximity to residential areas, and proximity to ground water. Two methods of dry and wet cooling were evaluated. According to the results, it was found that the favorable sites for the installation of wet cooling systems represent, respectively, 11.7 % and 5.5 % of the total area for dry cooling systems [36]. The potential of large-scale wind, solar photovoltaic, and CSP energies was studied by Anwarzai and K. Nagasaka, using multi-criteria decision analysis (MCDM) and geographic information systems (GIS) methods, taking into account resources, topography, environmental, and economic aspects. Based on the findings, it is estimated that the annual production amounts to 342,521 GWh of solar energy, 140,982 GWh of photovoltaic energy, and about 6000 GWh of concentrated solar power (CSP) technologies. This amounts to about 160 times the current electrical consumption [43]. Additionally, a GIS-AHP approach was used to locate solar PV and CSP plants throughout the Mediterranean region. The developed approach is based on technological, economic, and socio-environmental criteria, taking into account the various viewpoints of various stakeholders and allowing for the evaluation of various scenarios [34]. Furthermore, Fotsing Metegam I.F et al., applied the boolean method and the GIS to represent the optimal places for large-scale solar PV and CSP parks in Cameroon. Based on the results, the percentages of land that can be used to set up PV and CSP solar parks are roughly 10.17 % (on a 47,331,18 km^2^ area) and 0.56 percent (on a 2606,24 km^2^ area) [6].

Literature review on Concentrated Solar Power (CSP) indicates that sub-Saharan Africa, and Cameroon in particular, have been the subject of very few studies. The methodology employed for site selection in Cameroon, which combines Geographic Information Systems (GIS) with a Boolean method, is suboptimal, leading to less reliable results. Consequently, there has been no research that integrates multicriteria analysis with GIS within Cameroon's context. Therefore, by identifying the most suitable sites for CSP projects, investors and the government could significantly enhance the return on their investments. This study seeks to bridge the existing gap by examining the potential to enhance the reliability of technical, financial, and material input parameters via sensitivity analysis. This approach will provide a comprehensive range of options and a broad perspective. The results can be further refined by incorporating the population density factor. Moreover, the precision of the results can be improved by omitting areas less than 2.2 km^2^, in line with the regulations for large-scale CSP solar parks. Additionally, conducting a sensitivity analysis that either concentrates on the weights of technical and economic criteria or equalizes all criteria weights could reveal the effects of weight variability on the outcomes. The objective of this research is to assist stakeholders in identifying optimal locations for large-scale CSP solar energy installations using wet cooling, by integrating a Multi-Criteria Decision-Making (MCDM) approach with a Geographic Information System (AHP-GIS). This research contributes additional insights to the existing body of knowledge in this field.

- The first study's introduction is to investigate the suitability of solar energy (CSP) for large-scale humidification and its connection to the current electrical grid in Cameroon, based on a hybrid GIS-AHP approach at the national level.
- The provision of high-resolution (1 km × 1 km) solar energy adequacy cards for Cameroon, which are the highest-resolution available among all large-scale solar energy adequacy cards now available in the country,
- The development of a methodology based on in-depth knowledge of the local Cameroonian context by consulted experts, with the potential for transferability to other nations.
- The incorporation of population density parameters and required surface areas enables the study's results to be refined.
- A methodology that can be easily applied to other renewable energy technologies, such as solar photovoltaic (PV), wind energy, hydroelectric power, or biomass, once the criteria defining each technology have been carefully determined.
- The obtained results will enable national and international stakeholders to be informed about the best locations for the installation of CSP solar plants in Cameroon.

This article will begin with an introduction in 1, follow the presentation of the data in 2, and the methodology in 3, in 4, we will present and discuss the results obtained. Section 5 will present the conclusion of this study.

## 2 Data

### 2.1 Study area

Located at the bottom of the Gulf of Guinea, Cameroon is a country in central Africa, between the 2nd and 13th degrees of north latitude and the 9th and 16th degrees of east longitude. The area of the country is approximately 475,650 square kilometers. It has a maritime border of 420 km to the southwest along the Atlantic Ocean. Its western border is Nigeria; its southern border is Congo, Gabon, and Equatorial Guinea; its eastern border is the Central African Republic; and its northeast border is with Chad. Finally, at the highest point of the triangle, to the north, it is surrounded by Lake Chad [44]. Cameroon has a varied natural environment. This country is considered Africa in miniature. Indeed, various categories of natural zones play a role in the geographical variety of the country. The maritime and equatorial zones are occupied by the forested south (Central, Eastern, Littoral, South, and South-West provinces). This region is distinguished by its abundant vegetation, its vast hydrographic network, and its hot and humid climate with abundant precipitation [44]. The high plateaus of the west (West and North-West region), including average altitudes exceeding 1100 m, constitute an agro-volcanic zone (coffee, market gardeners, etc.). The Western highlands (West and North-West regions), whose average altitude exceeds 1100 m, constitute an agro-volcanic zone (coffee, market gardeners, etc.). The North Sudano-Sahelian (Adamawa, North, and Extreme North) is an area of savannahs and steppes. Despite the more temperate climate of the Adamawa plateau, the rest of this region is marked by a hot and dry tropical climate with increasingly low precipitation the closer we get to Lake Chad [44].

**Fig. 1:**
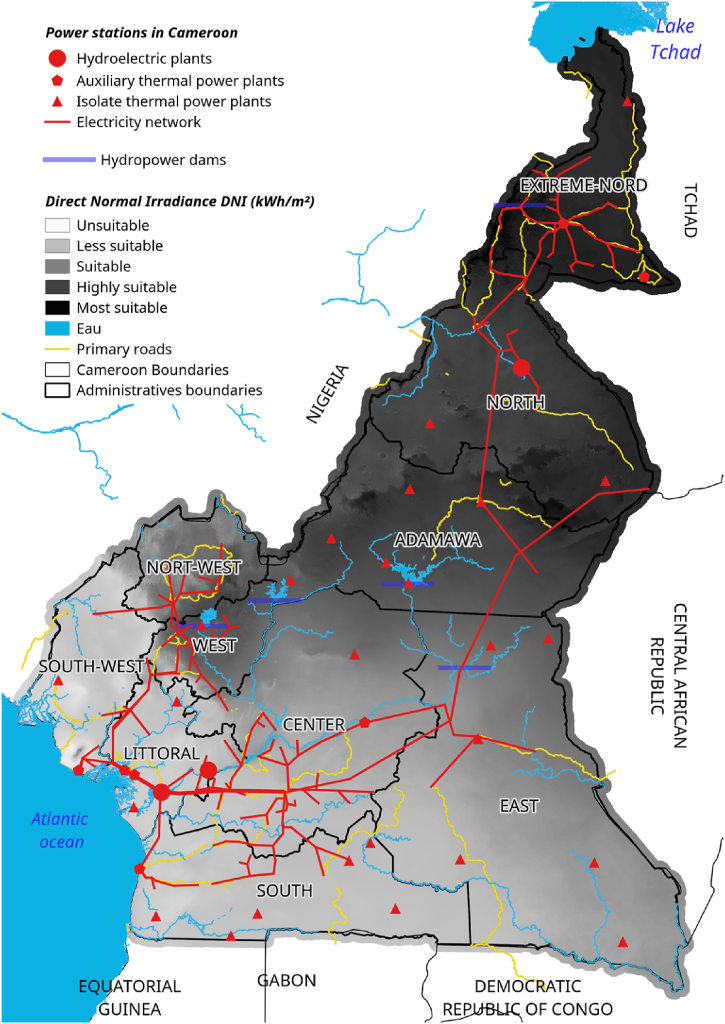
Cameroon energy map.

Three hydroelectric power plants supply the RIS, while nine thermal power plants are connected to the network, eight (8) of which flow into the RIS. Several isolated power plants are also identified, providing power to the RIS and RIN regions and the Eastern network zone [45]. In this way, the large southern area concentrates more than 56 % of the electricity fleet, with 83 % of the main power plants connected to the network and 44 % of the isolated power plants. This should demonstrate the relative importance of demand from the Great South network. On average, isolated thermal power plants represent 1.6 % of ENEO's national production, with a peak of 1.8 % in 2011, according to Bank World. The energy map of Cameroon is illustrated in Fig. 1 [46]. Table 3 presents the description of the data used in this study, as well as the format used and the different sources of this data.

**Table 3:** Description of study data.

Data	Types (format)	Geometry/resolution	Sources
Administrative limits of Cameroon (regions, divisions, districts) Map	Vector (shapefile)	Polygon	GADM,2022 [47]⁠
Annual Direct Normal Irradiance (DNI) in kWh/m^2^	Raster	(1∗1 km^2^)	**Solargis, 2022** [48]**⁠**
Map of population density in Cameroon	Raster	(1∗1ha)	Wordpop, 2010 [49]⁠
Map of Cameroon Power lines	Vector (shapefile)	Point	World Bank [50]⁠
Hydrological map of Cameroon (streams, navigable waters, rivers, rivers, wetlands, reservoirs …).	Vector (shapefile)	Line, polygon	OSM,2022 [51]⁠
Map of land use in Cameroon	Vector (shapefile)	Polygon	OSM,2022 [51]⁠
Map of the road network (inter_state, primary, secondary roads …) and railways in Cameroon.	Vector (shapefile)	Lines	OSM,2022 [51]⁠
Map of elevation in Cameroon	Raster	(1∗1 km^2^)	Global Wind Atlas [52]⁠⁠
Temperature (°C)	Raster	(1∗1 km^2^)	Solargis, 2022 [48]⁠⁠
Map of Cameroon airport	Vector (text format csv) Geometry	Point	ADC (Cameroon airport), 2022 [53]⁠

**Table 4:** The fundamental scale of absolute numbers [54]⁠⁠

Intensity of Importance	Definition	Explanation
1	Equal Importance	Two activities contribute equally to the objective
2	Weak or slight	
3	Moderate importance	Experience and judgment slightly favor one activity over another
4	Moderate plus	
5	Strong importance	Experience and judgment strongly favor one activity over another
6	Strong plus	
7	Very strong or demonstrated importance	An activity is favored very strongly over another; its dominance demonstrated in practice
8	Very, very strong	
9	Extreme importance	The evidence favoring one activity over another is of the highest possible order of affirmation

**Table 5:** Random index RI.

n	1	2	3	4	5	6	7	8	9	10
RI	0,00	0,00	0,058	0,90	1,12	1,24	1,32	1,41	1,45	**1,49**

### 2.2 Solar power potentials and CSP technology

There are four categories of solar production potential: geographic, technical, economic, and environmental. We can define the geographic potential of solar production in a given area as the total quantity of annual solar radiation available in this area, taking into account the constraints on the geographic potential of an area (e.g., land covered by forests or bodies of water). The technical potential of solar production in a specific area can be defined as the amount of geographic potential that can be converted into electricity, taking into account available solar energy technologies. Economic potential refers to the technical possibilities that could be realized at a price equivalent to conventional electricity sources. And the environmental potential allows us to protect our environment by respecting environmental criteria. In 1979, Sandia National Laboratory established the world's first commercial CSP (concentrated solar power) plant in New Mexico. Since then, the United States and Spain have been the countries that have invested the most in solar power. CSP power plants, with the majority of the world's installed electrical power using this technology (515 and 1002 MWe, respectively). Four major CSP technologies are present: the disk, the parabolic trough, the Fresnel, and the concentration tower. The largest in the world is located in California, with an electrical power of 150 MW. Several projects are currently under construction (more than 200 MW in Spain, and new projects represent more than 2000 MW). In addition, CSP solar technology is the most likely for the massive implementation of solar power in North Africa. With this in mind, Algeria has concluded a partnership agreement with the German Solar Jülich Institute to build a solar tower [2].⁠. The first large-scale solar thermal power plant, Solar Engine One, was built in 1913 by Frank Shuman and C.V. in Egypt to pump water [3]⁠. The most advanced industrial technique is concentration using parabolic trough mirrors. These types of mirrors focus on a receiving tube, which contains a fluid containing absorbed heat. Then the liquid produces vapor, which is then turbined to generate electricity. Another type of thermodynamic power plant consists of hundreds of mirrors (heliostats), which make it possible to converge solar radiation on a high tower where a boiler is installed. This type of installation has a power output of between a few megawatts and a hundred MW [33]. Concentrated solar power (CSP) technology uses mirrors that transform solar energy into heat, which is then converted into electricity using steam turbines, gas turbines, or stirring engines. The CSP sector is booming, particularly in Europe (Spain is in the lead) and the United States. Among the different forms of CSP power plants, we find parabolic tanks, power towers, satellite dish systems, and FRESNEL tank technology. The investment costs, land requirements, and efficiencies of these technologies vary [33]. The CSP market is dominated by power systems. parabolic trough. In 2010, CSP plants in operation and under construction used parabolic trough technology, while planned CSP plants accounted for 75 %. Reported “solar-electricity” efficiencies of CSP technologies show disparities. The efficiency of CSP power plants was mentioned by Ref. [37]⁠: the parabolic trough represents 15–21 %, the electrical tower 18–20 %, and the parabolic antenna 25–30 %. In 2022, global concentrated solar thermal (CSP) capacity increased by 200 MW, reaching a total of 6.3 GW. This increase is the consequence of the first year of a decline in global solar capacity. CSP in 2021. Overall, global CSP growth has slowed after an initial expansion in Spain and the United States in the early 2010s. 3 None of these major markets have added capacity in nearly a decade. However, new initiatives have been released and are being implemented in developing markets, such as Chile, China, Israel, Morocco, South Africa, and the United Arab Emirates.

Thermal Energy Storage (TES) systems store thermal energy, either heat or cold, for future use. This approach is vital for optimizing the use of energy resources, enhancing energy efficiency, and integrating renewable energies into the grid more effectively [3]⁠. TES is instrumental in increasing the proportion of renewable energy, thus aiding in decarbonizing critical sectors like power [3]⁠. In 2020, the global TES capacity was around 234 GWh, and it is projected to reach 800 GWh by 2030, with molten salt storage capacities leading at 21 GW h [4]⁠. Electric heating systems are being explored for directly heating molten salts with electricity from renewable sources like photovoltaic panels. The solar tower represents the pinnacle of Concentrated Solar Power (CSP) technology, featuring a field of heliostats—flat mirrors that follow the sun and focus its rays onto a solar receiver. This advanced heat exchanger allows the heat transfer fluid (water, molten salt, or solid particles) to reach extremely high temperatures, leading to greater efficiencies [4]⁠. More advanced than earlier CSP technologies, the solar tower can achieve temperatures of 565 °C with molten salts and aims to surpass 800 °C using solid particles [4]⁠. For thermal energy storage, CSP plants utilize molten salts, a medium that is low-cost, non-flammable, and environmentally friendly. There are various forms of thermal energy storage. Sensitive storage increases the temperature of a material, like water or molten salt, for energy retention. Latent storage leverages the physical transitions, such as melting and solidification, to store and release energy using phase change materials (PCMs). Thermochemical storage employs reversible chemical reactions for energy storage and retrieval, enabling a higher density of storage. These methods enhance energy efficiency in building climate control systems, solar thermal power stations, and industrial procedures [3]⁠ [4].

### 2.3 Data

The optimal deployment of a CSP solar farm requires considering several criteria and factors aimed at optimizing the location of CSP parks. This will make it possible to have a more efficient, and economical system with less impact on the environment. Decision criteria are typically derived based on the study objective, data accessibility, and existing literature. Furthermore, most CSP solar site suitability studies consider DNI solar irradiation as the most important decision criterion, as shown in Table 2 [41]. This study will use the MCDM technique combined with GIS analysis to evaluate suitable sites for large-scale CSP plant installations in Cameroon. The GIS-MCDM-AHP approach generally has three essential steps [29]. It generally begins with the extraction of unsuitable layers based on restrictive criteria using different GIS databases at high spatial resolution. Subsequently, evaluation criteria with three criteria and ten sub-criteria for the installation of CSP plants are proposed, and the AHP method is used to calculate their weightings. Finally, land suitability maps are established using GIS tools, and the theoretical and technical potentials of CSP systems are estimated with a sensitivity analysis. The data sources as well as the format type and geometry of the data used in this study are listed in Table 3. The resolution used in this study is 1∗ 1 km^2^.

#### 2.3.1 Exclusion criteria

Inappropriate areas limited by climatic, orographic, location, and watercourse factors should be removed from the initial map layer by the GIS. Through a comprehensive analysis of the geographic coverage of the study area and an in-depth review of relevant literature [33,41,26,30,30,17,[55], [56], [57], [58]], the following exclusion criteria are desirable: protected area, land use, transport infrastructure, airports, waterways and orography. Table 6 summarizes the constraint variables with a detailed explanation. We will also exclude areas such as transmission lines, roads, and cities, as well as buffer zones around these areas. Furthermore, areas that present a technical limitation such as areas with land with a steep slope (>7 %), an elevation less than 1500m [7], and an annual solar irradiation DNI of 1500kwh/km^2^ are also excluded. Also excluded are remote areas that have a distance of more than 30 km from power lines, more than 10 km from roads [7], more than 45 km from residential areas, and areas with more than 500 inhabitants/km^2^ [33]⁠. Table 6 provides an overview of the excluded areas, their buffer zone, and maximum distances. We will use a Boolean method combined with GIS to extract the final map of constraints presented in Fig. 5. 0 will be assigned to inappropriate zones and 1 to appropriate zones.

**Table 6:** Restriction criteria from chosen solar farm and CSP site selection studies [6]⁠

Restriction criteria	Plant required area CSP	DNI (Annual direct normal irradiation)	Temperature	Buffer distance/proximity from electricity grid⁠	Buffer distance from airports, m []	Proximity /buffer to roads & highways⁠	Buffer distance from Protect area and land-use (forests & parks …)			Population density, Minimize density		Buffer distance/proximity from residential	Buffer distance from streams⁠	Buffer distance from rivers⁠	Slope⁠	Elevation ⁠
Values	≥2,2 km^2^ [43]^⁠^	≥1500 kWh/m^2^ [7]⁠⁠⁠	≥20° [59]⁠	>250 m and ≤30 km [7]⁠	>3500	>500 m and ≤10 km [7]⁠	Not within protect area (buffer 500m) [7]:⁠			≤500 inhabitants/km^2^(5 inhabitants ha) [23]^⁠^	>2 km and <45 km [2]⁠⁠		≥100 m and ≤10 km [43]⁠	⁠≥100 m and ≤10 km [43]⁠	<2 % (1,15°) [23]⁠	>1500m [60]

#### 2.3.2 Evaluation criteria

**Fig. 2:**
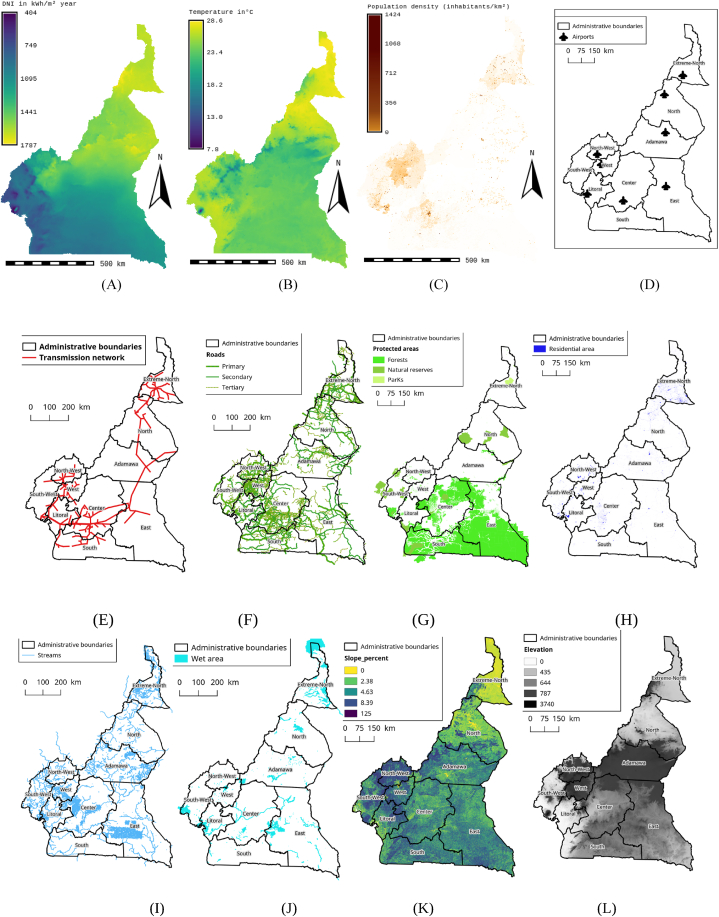
Evaluation Data (A) GHI, (B) Temperature, (C) Population density, (D) Airports, (E) Electricity grid, (F) Roads, (G) Land use, (H) Residential Areas, (I) Streams, (J) Wet areas, (K) Slope (L) Elevation.

The evaluation criteria vary depending on the desired objective, the study area, the accessibility of the datasets, the spatial scale, and the opinion of experts in the field consulted during the study. The criteria chosen are based on a careful analysis of the literature, summarized in Table 1, Table 2 The current research is carried out based on the already-defined criteria. Three main categories are selected for the evaluation criteria: technical, socio-environmental, and economic. Fig. 2 presents the sub-factors that are part of these main categories. The next part addresses and develops the criteria taken into account according to their importance.

a) CSP (climate factor) solar resources

DNI solar irradiation is the essential factor for choosing a large-scale CSP solar farm [61]. Several studies have used solar irradiation DNI as the main factor for the choice of solar farms [2,6,[29], [32], [33], [36], [37], [38], [42], [62],^63^]⁠. To ensure that the selected sites will be competitive in the solar market, it must have a significant amount of DNI in the region and this condition presents a geographic restriction in the selection process. The efficiency of CSP power plants strongly depends on the level of solar irradiance. The direct normal irradiation (DNI) potential is the most important factor for CSP site selection because it directly influences the operational performance of the plant and the feasibility of projects. One of the advantages of CSP technology is the presence of a thermal intermediate, which offers possibilities for thermal storage and hybridization with fossil fuels [64]. Disadvantages include high land and water requirements (unless dry cooling). In this study, we used 1500kwh/km^2^ as unsuitable, 1500–1550 kWh/km^2^ as “less suitable”, 1550–1600 kWh/km^2^ as “suitable”, 1600-1700kwh/km^2^ “highly suitable”, and 1700–1787 kWh/km^2^ as “most suitable” as L. Sun et al. [29],⁠. The DNI solar irradiation data comes from Solargis, 2022 [52]. Fig. 2-A presents DNI solar irradiation in Cameroon.

b) Distance from the electricity network (location factor)

Being close to the power grid plays a vital role in setting up CSP solar power installations on a wide-area grid. (12–15), (17–19). When solar energy is not enough, grid connectivity can simplify the supply of energy to electrical equipment in the system. Because the development of new energy transmission systems involves high costs, it is better to use the existing power transmission line to decrease the capital expenditure of the project. To achieve this goal, the CSP solar power plant must be as close to the power grid as possible. This also helps decrease power losses caused by long transmission line distances and avoids the need to build a costly new power plant. For this analysis, the maps of the national electricity network and the power plant are obtained from the electrical data register of the national electricity company ENEO, which manages electrical energy in Cameroon. In this research, 10 km around the electricity network was chosen as a favorable area for the installation of CSP solar parks [7]. However, there is a safety distance of 250 m between the CSP solar park and the electricity lines [7]. Information regarding the electricity network was taken from the World Bank website in 2018 [50]. Fig. 2-E present electricity network map of Cameroon.

c) Distance from residences (location factor)

The distance between residential areas plays a crucial role in the placement of CSP solar installations [1,2,42,62,29,36,23].All residential areas and areas located less than 2 km from residential areas are not suitable for the establishment of a solar park. Given the various adverse environmental effects of urban centers and expanding residential areas, distance from residential areas is seen as one of the key criteria in choosing solar sites for CSP. In addition, areas located more than 45 km from urban centers are perceived as areas not very conducive to the creation of solar farms [2]. These distances are clearly defined in all similar studies. However, these buffer distances vary between studies and must be justified individually by the national planning authority. Solar parks must be located at a reasonable distance from settlement areas to reduce the maximum transmission losses. Information concerning residential areas comes from the Open Street Map (OSM, 2018) [51]. We nevertheless observe a safety distance of 2 km around residential areas. Fig. 2-H present residential map of Cameroon.

d) Distance from roads (location factor)

Different researchers have considered the distance from the road network as a crucial factor in choosing favorable sites for CSP solar. [2], 12–15, 17–19, and 61. For the construction and maintenance of CSP solar farms, it is essential to have access to vehicles. It is considered that the proximity of a CSP solar plant to an existing road is an economic element. This helps to avoid additional expenses linked to the construction of a road and, above all, to prevent the harmful effects on the environment that building a new road would cause. Building a large-scale solar farm in inaccessible areas will result in a significant increase in capital expenditure, which will incur additional costs compared to the initial investment [65]. A distance of 10 km around the roads was chosen as a favorable area for the installation of CSP solar power plants. However, there is a safety buffer of 500 m between the solar park and the road network. Information concerning the roads is taken from the Open Street Map 2018 [51]. Fig. 2-F present main roads map of Cameroon.

e) Population density (location factor)

Population density plays a crucial role in establishing favorable sites for the use of solar CSP [32]. Because electrical energy cannot be stored and causes considerable losses during its transport, it would be preferable to install large-scale CSP solar installations near densely populated areas to minimize gasoline transportation losses. However, the risks posed by CSP solar farms require that they be built away from areas with high population density to preserve the population. In this research, areas where the population is less than 500 inhabitants per km^2^, or less than 5 inhabitants per hectare, are considered favorable sites for large-scale CSP plants [33]. The map illustrating population density comes from WorldPop [49]. Fig. 2-C present density population map of Cameroon.

f) Slope (orography factor)

Slope plays a crucial role in identifying favorable sites for the installation of CSP solar power plants [1,2,42,62,[29], [32], [36],^23^,^43^]. The installation and maintenance of solar parks are limited by the significant slope of the land. According to the studies, the maximum allowable slope for CSP solar farms ranges from 0.5 to 3 %. Topography with steep slopes is often deemed unsuitable for solar farm development due to the additional costs associated with construction and maintenance. The slope of 2 % was considered a maximum threshold for CSP plants in this study [22].⁠ [23]⁠. The elevation map that allows the slope to be determined is extracted from data from the Global Wind Atlas [66]. Fig. 2-K present slope map of Cameroon.

g) Land use (constraint factor)

It is essential to carry out a thorough assessment of available land when setting up an energy project. Large-scale CSP solar farms should be located outside protected areas to minimize the negative impact on the environment [32,30,30,31,56,[60], [67], [68]]⁠. Protected areas in this study include parks, forests, and nature reserves. Information concerning land use is taken from OSM 2018 [51]. Fig. 2-D present protected areas map of Cameroon.

h) Distance from airports (constraint factor)

In many studies, a safety distance is observed around airports. This is also a crucial element due to the harmful consequences of CSP solar parks on aviation activities, such as disruption of pilot vision, risks of disasters, etc. Thus, it is essential to place large-scale CSP solar parks at a significant distance from airports. This is the reason why we observe a buffer zone of 2500 m around airports [10]. Information regarding the location of airports comes from Ref. [53]. Fig. 2-D present airport map of Cameroon.

i) Elevation (orography factor)

Elevation plays a crucial role in selecting favorable sites for the installation of CSP solar. [2], 12–15, 17–19, and 61⁠. In the physical realm, solar parks must be located in suitable locations. In general, high-altitude regions are perceived as less suitable due to additional construction and maintenance costs. In this study, a maximum altitude of 1500 m was considered a threshold [7]. The graphic representation comes from Global Atlas Wind [66]. Fig. 2-L present elevation map of Cameroon.

j) Plant required

The minimum area required plays a crucial role in the installation of CSP solar sites. It is important to emphasize that we are setting up large-scale CSP solar plants rather than solar panels to power a building. Connecting these solar plants to the national electricity grid will increase electricity production. In this study, it is necessary to have a minimum area of 2.2 km^2^ to install a large-scale CSP solar park [35,69].

k) Temperature (climate factor)

One of the indispensable elements of CSP systems is the solar panel. To provide the necessary energy, the system must be designed adequately. Efficiency is one of the issues that impact the determination of the size of the panel needed. Solar panels are very efficient when their temperature is high, and the panel temperature is influenced by the ambient temperature and the intensity of solar radiation. The modules of CSP systems lose their performance when the ambient temperature increases. Cell temperature increases by 1 °C at temperatures above 25 °C, while power production decreases by approximately 0.4 %–0.5 % [37]. Fig. 2-B present temperature map of Cameroon.

l) Distance from watercourses (Watercourse factor)

**S**tudy will consider parabolic trough CSP technology which uses thermal energy generated by the solar field to drive a steam turbine. The water requirements for cooling of such a CSP plant are similar to those of any plant based on the Rankine cycle. The type of cooling is wet cooling which requires large amounts of water for condenser cooling. It is therefore strongly recommended to build large-scale CSP plants near water sources. In this study, the eligible water sources to supply wet-cooled CSP plants are Watercourses, rivers, and streams. Furthermore, large-scale CSP solar farms can contaminate or pollute aquifers such as permanent water bodies, reservoirs, dams, lakes, and rivers [60]⁠. To do this, we have created a safety zone (buffer) of 100 m [35]⁠ around the watercourses to avoid their pollution. However, during their operation, all CSP solar power plants need water to clean dust from the panels. It should be noted that dust is a constraint factor for the efficiency of the panels. Several authors have considered proximity to waterways as an essential factor when choosing the location of a large-scale CSP power plant. We can thus cite [2,42,62,[29], [32], [36],^23^,^43^]⁠. Fig. 2-I and 2-J present watercourses map of Cameroon.

The spatial distribution of the different input data for this study is illustrated in Fig. 2.

## 3 Methodology

To successfully carry out this study, a seven-step analysis was carried out to improve the selection of favorable sites for solar plant selection CSP-grid-connect.

• Step 1: Determination of the different layers of thematic constraints to eliminate inappropriate areas using a boolean method coupled with GIS.
• Step 2: Implementation of the AHP approach to determine the weight of the various study criteria.
• Step 3: Reduction of input parameters for different layers
• Step 4: Creation of final suitability maps
• Step 5: Evaluation of sensitivity by adjusting factor weights (scenario testing)
• Step 6: Evaluation of the theoretical solar technical potential.
• Step 7: Technical and geographical assessment of the ten regions of Cameroon.

**Fig. 3:**
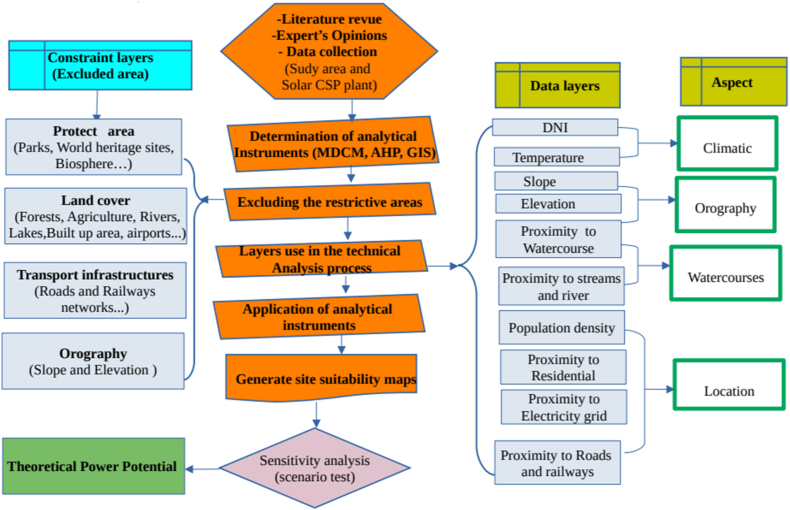
The steps for identifying suitable sites for large-scale solar CSP farms.

The methodological framework, presented in Fig. 3, outlines the steps for identifying suitable sites for large-scale solar CSP farms, including site selection criteria, data analysis, and final determination.

### 3.1 Geographic Information Systems (GIS)

GIS is a powerful tool for viewing, analyzing, and modifying data, maps, and spatial information. It is also an excellent tool for the strategic planning of solar energy development projects due to its high maturity and advanced embedded tools. Developing a decision support model that incorporates a GIS-MCDM can help identify optimal locations for solar power plants connected to the existing power grid. Thus, it is crucial to improve the performance of solar projects to maximize the power output produced while reducing project costs. The integration of GIS with other techniques develops a better understanding for decision-makers so that they can optimize their options, taking into account various subjective and contradictory criteria.

GIS was created for the purpose of collecting, storing, manipulating, analyzing and representing geographic information [70]. GIS uses two types of coverage representation: rasters and vectors. The rectangular grid of the raster is known as a pixel and contains precise data based on a specific geographic location. Vectors manage a geometric figure (points, lines and polygons) which establishes the limits linked to a coordinate system. The duration of this data is defined in a geodatabase, which ensures the organization and standardization of the data. In this study, all geographic data were processed using rasters due to the continuous analysis of spatial variables. The vector data was transformed into raster, recoded according to Table 6, Table 7, then grouped as follows: The value of “Unsuitable” areas is 0, “Less suitable” areas are 1, “Suitable” areas are 2, “Highly suitable” areas are 3, and “Most suitable” areas are 4. The MCDM-AHP method in a GIS environment is based on criteria represented by a layer of geo-referenced cartographic information [71]. The free software GRASS GIS.7 [72] was used to rasterize and normalize the data layers in this research. With this starting point and the generated inputs, the statistical calculations and layout of the figures were also done in this same software.

**Table 7:** Evaluation criteria and their reclassification with the corresponding scores of large-scale grid-connected CSP sources [2,2,38,42,33,32,36,73]:⁠⁠⁠

Aspect		Climate		Orography		Location				Water resource	
Score	Land suitability index (grade)	Annual direct normal irradiation (DNI) in kWh/m^2^ year	Temperature in °C	Elevation in m⁠	Slope⁠	Proximity to roads and railways in m⁠	proximity from electricity grid in m	proximity from residential in m	Population density, Minimize density in inhabitants/km^2^	Proximity to watercourses in m⁠	Proximity to streams and rivers in m⁠
0	Unsuitable	<1500	<20	>1500	>7	<500 and >10000	<250 and >30000	<2000 and >45,000	>600	<250 and >30000	<250 and >30000
1	Less suitable	1500–1550	20–22	1100–1500	6–7	>5000	>20,000	20000–45000	500–600	>3000	>3000
2	Suitable	1550–1600	22–24	700–1100	5–6	3000–5000	10000–20000	10000–20000	100–500	1000–3000	1000–3000
3	Highly suitable	1600–1700	24–26	300–700	3–5	1000–3000	5000–10000	6000–10000	0.0001–100	500–1000	500–1000
4	Most suitable	1700–1787	≥26	<300	<3	500–1000	250–5000	2000–6000	0	250–500	250–500
Land suitability CR = 5,85 %	Weight	0,2184	0,045	0,203	0,063	0,143	0,101	0,099	0,066	0,031	0,025
	Nornalized weight	22 %	20 %	7 %	3 %	5 %	14 %	3 %	6 %	10 %	10 %
		42 %		10 %		28 %				20 %	
Scenario 1 (Technical weight) CR = 2,5 %	Weight	0,588		0,104		0,053				0,255	
	Nornalized weight	59 %		10 %		5 %				26 %	
Scenario 2 (Economic CR = 4,2 %	Weight	0,256		0,051		0,576				0,117	
	Nornalized weight	26 %		5 %		57 %				12 %	
Scenario 3 (Equal-Weight)	Weight	20 %		20 %		40 %				20 %	

### 3.2 Analytical hierarchy process (AHP)

The AHP method is a mathematical and psychological method used to measure the weight of criteria using a series of pairwise comparisons [74]. Furthermore, another captivating feature of the AHP method is its ability to ensure consistency in decision-making, thereby reducing bias in the decision-making process. This study uses the AHP method to assess the importance of the evaluation criteria for CSP solar energy installation. Different researchers have used AHP to identify favorable locations for establishing solar farms in various countries [19,26,27,28,13,17,18,58,59,52,[73], [75], [76], [77], [78], [79]]⁠. Hierarchical Analysis Process (AHP) is a method used to analyze pairwise comparisons. It provides a basic numerical scale ranging from 1 to 9 and the ability to measure the quantitative and qualitative performance of priorities [80]. The fundamental scale of absolute numbers Saaty is illustrated in Table 4. A structured decision-making approach using expert judgments is as follows [55][81]⁠

1. Description of the problem
2. Elaboration and hierarchical structuring of the problem at various stages to identify the objectives and outcomes of the problem based on the objective, criteria, and options.
3. A numerical pairwise comparison scale is used to weight each criterion, as shown in Table 4.
4. Perform calculations to determine the maximum eigenvalue *λ*_max_, the consistency index CI provided by Equation (1), the consistency ratio (CR) provided by Equation (2), and the normalized values for each criterion.

(1) $$CI=\frac{\lambda_{\max}-n}{n-1}$$

where n is the number of criteria and *λ*_max_ is the pairwise comparison matrix's eigenvalue.

The consistency ratio must be determined in order to assess the consistency of the decision-maker's paired scores.

(2) $$CR=\frac{CI}{RI}$$

Where RI is the random index that corresponds to the number of criteria that are not repeated in Table 5 and CI is the coherence index provided by Equation (1). If a matrix exists with a value of CR or an unacceptable composite weight, that is, CR > 0.10, the expert is required to make multiple judgments on this matrix until the values reach the desired level. Calculating the land suitability indices S yields the global suitability score following Equation (3):

(3) $$S=\sum_{i=1}^{i=N}W_{i}*P_{i}$$

Where W_i_ represents the weight of the second criterion and Pi represents the score of the second factor. The assessment of the solar energy production potential of connected and off-grid photovoltaic systems is based on the factors and weights selected in Table 6, Table 7 These two tables also present the weightings corresponding to the sensitivity analysis. We obtain the final map by multiplying the map of Equation (3) by the constraint layer: that is to say Equation (4) which follows.

(4) $$SuitabilityMap=\left(\sum_{i=1}^{i=N}W_{i}*P_{i}\right)*Constraint$$

### 3.3 Calculation of the CSP technical potential

The theoretical potential of the CSP depends on the availability of the solar resource when used alone. Biophysical and socio-economic elements modify the theoretical potential to create the geographical potential. After the start-up of the CSP plant, other elements such as capacity, cooling technology, and electricity cost are taken into account, which further reduces the value to assess the technical economic potential. It is complex to determine the value of the latter potential because it is difficult to determine the capacity factor until the plant is in full operation. Thus, the realistic design of a PTC-type CSP plant is based on the technical potential, which includes land availability and accessibility, solar-electric efficiency, and the desired configuration of the plant. A calculation of the CSP potential in Africa was made using a simplified basic mathematical formula that only requires the DNI, total available area, and land use factor. According to the recommended formula, the annual CSP technical potential (in kWh/y) of a PTC plant is calculated as a function of the local solar resource Ω, defined by the average annual DNI (in kWh/m^2^y-^1^), multiplied by the total land use area Y (in m^2^), land use efficiency θ, and overall annual production of the facility due to dry cooling θ. Thus, it is possible to calculate this technical potential according to the following formula in Equation (5):

(5) CSP=Ω∗Y∗θ∗δ

This study considers a CSP park efficiency of 17.9 % [32]⁠ and land use efficiency is the product of the land use factor (0.4) [38]. The study took into account the intensity of solar light (DNI) as the average for each range. For the “Less suitable” range, we have 1525 kWh/m^2^y-^1^.year; for the “Suitable” range, we have 1575 kWh/m^2^y-^1^; for the “Highly suitable” range, we have 1650 kWh/m^2^y-^1^; and for the “Most suitable” range, we have 1700 kWh/m^2^y-^1^. This distribution gives closer results and exact values.

## 4 Results and discussions

**Fig. 4:**
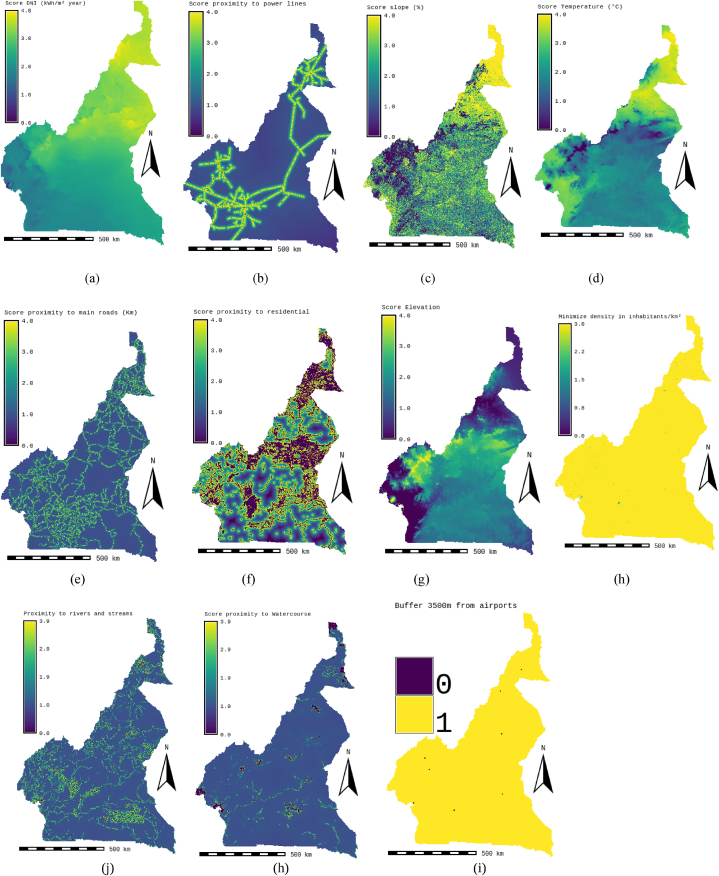
Score of location factors of solar CSP systems (a) DNI, (b) Distance from electricity grid, (c) Slope, (d) Temperature, (e) Distance from Roads, (f) Distance from residential, (g) Elevation, (h) Population density, (j) Distance from streams, (h) Distance from watercourses, (i) Buffer around airports.

Identifying and finalizing the selection criteria for this study required an extensive literature review and approval from ten experts to reduce conflicts of interest and personal bias [9]. Professional engineers, managers of national energy authorities, university professors, and researchers with solid expertise in solar energy applications and knowledge of Cameroonian soil conditions are selected as experts. All these specialists work in Cameroon. Table 11 presents a list of experts from various organizations in Cameroon, detailing their designations, qualifications, age, work experience, and affiliated departments/companies. Scores The following land suitability indices were assigned: “0” for Unsuitable; “1” for Less Suitable; “2” for Suitable; “3” for Highly Suitable; and “4” for Most Suitable. With this, the ten factors of the study were reclassified. Fig. 4 shows the reclassified layer maps of the CSP-grid-connect plant location factors.

**Table 8:** Statistical information of land suitability areas for three sensitivity cases.

Plage	Categories	Statistics	AHP-GIS Grid-connect CSP	Technic CSP-grid-connect (Scenario 1)	Economic CSP-grid-connect (Scenario 2)	Equal-weights CSP-grid-connect (Scenario 3)
0–0	0	Areas in m^2^	244229834843,097	244229834843,097	244229834843,097	244229834843,097
		Pixel count	24421627	24421627	24421627	24421627
		%	42,35 %	42,35 %	42,35 %	42,35 %
0,001-1	1	Areas in m^2^	38852000	28821600,782	246683701,072	909230499,617
		Pixel count	3885	2882	24667	90918
		%	0,001	0,001	0,04 %	0,16 %
1001-2	2	Areas in m^2^	253773954942,223	216738977947,054	260793304810,665	308847513731,905
		Pixel count	25375986	21672694	26077882	30883036
		%	44,00 %	37,58 %	45,22 %	53,55 %
2001-3	3	Areas in m^2^	78661888964,735	115605850848,380	71247797182,866	22729612426,785
		Pixel count	7865752	11559943	7124384	2272835
		%	13,64 %	20,05 %	12,35 %	3,94 %
3001-4	4	Areas in m^2^	47652646,678	66733706,460	175929771,324	0
		Pixel count	4765	6673	17592	0
		%	0.01 %	0.01 %	0.03 %	0 %

**Table 9:** Theoretical solar power potential in TWh/year of solar CSP-grid-connect.

Scenario	Categories /Parameters	Not Suitable	Less Suitable	Suitable	Highly Suitable	Most Suitable	Total
	Mean GHI irradiation in kWh/m^2^/year	–	1525	1575	1650	1700	
CSP AHP-Grid-connected	Area in km^2^	244229,835	38,852	253773,955	78661,889	47,653	576752,332
	Potential in TWh/year	–	4242	28618,089	9293,116	5800	**37921,247**
	Potential in GW	–	0,484	3266,905	1060,858	0,662	4328,425
Scenario CSP-Technic grid-connected (Scenario 1)	Area in km^2^	244229,835	28,822	216738,978	115605,851	66,734	576670,22
	Potential in TWh/year	–	3147	24441,655	13657,675	8123	**38110,600**
	Potential in GW	–	0,359	2790,143	1559,095	0,927	4350,525
Scenario CSP-Economic grid-connected (Scenario 2)	Area in km^2^	244229,835	246,684	260793,305	71247,797	175,930	576693,551
	Potential in TWh/year	–	26,935	29409,661	8417,215	21,414	**37875,225**
	Potential in GW	–	3075	3357,267	960,869	2445	4323,656
Scenario CSP-Equal-weight grid-connected (Scenario 3)	Area in km^2^	244229,835	909,23	308847,514	22729,612	**-**	576716,191
	Potential in TWh/year	–	99,279	34828,734	2685,276	–	**37613,289**
	Potential in GW	–	11,333	3975,883	306,538	–	4293,754

**Table 10:** Theoretical solar power potential solar CSP-grid-connect per region in Cameroon (TWh/year).

		Unsuitable	Less suitable		Suitable		High suitable		Most suitable		Total				
		Mean DNI in kWh/m^2^/year	1525		1575		1650		1700						
**N°**	**Regions**	**Area Csp in km**^**2**^	**Area Csp in km**^**2**^	**Potential in TWh/year**	**Area Csp in km**^**2**^	**Potential in TWh/year**	**Area Csp in km**^**2**^	**Potential in TWh/year**	**Area Csp in km**^**2**^	**Potential in TWh/year**	**Area Csp in km**^**2**^	**Suitable Area in km**^**2**^	**Potential in TWh/year**	**Potential in TWh/year/km**^**2**^	**% Suitable area**
1	**Adamawa**	7612	13395	1462,60	57575	6492,733	118	13,94	–	–	78700	71088	7969,273	0,101	90,33 %
2	**Central**	43986	6263	683,86	36603	4127,720	16	1,89	–	–	86868	42882	4813,468	0,055	49,36 %
3	**East**	101632	7758	847,10	22495	2536,761	–	–	–	–	131885	30253	3383,857	0,026	22,94 %
4	**Extreme-North**	8645	–	–	5031	567,346	26860	3.173,24	40	4869	40536	31931	3745,46	0,092	78,77 %
5	**Litoral**	6663	941	102,75	18326	2066,623	60	7,09	7,6	0,925	25990	19334,6	2177,38	0,084	74,39 %
6	**North**	11873	489	53,39	61267	6909,080	6445	761,41	–	–	80074	68201	7723,886	0,096	85,17 %
7	**North-West**	1664	4430	483,71	16404	1849,879	–	–	–	–	22498	20834	2333,591	0,104	92,60 %
8	**West**	2963	1621	177,00	13317	1501,7581	5	0,59	–	–	17906	14943	1679,346	0,094	83,45 %
9	**South**	49292	1487	162,37	9022	1017,411	43	5,08	–	–	59844	10552	1184,856	0,020	17,63 %
10	**South-West**	9871	2468	269,48	20035	2259,347	17	2,01	–	–	32391	22520	2530,836	0,078	69,53 %
Total		244201	38852	4242,25	260075	29328,658	33564	3.965,25	47,6	5,793872	576692	332491	37536,159	0,065	57,65 %

**Table 11:** List of experts.

N°	Designation	Qualification	Age	Work experience	Department/Company
1	Professor	PhD	36	12	University of Dschang, Cameroon
2	Professor	PhD	50	25	University of Dschang, Cameroon
3	Assistant-Manager	PhD	43	20	Ministry of energy, Cameroon
4	Energy expert	Graduate	48	23	Ministry of energy, Cameroon
5	Energy expert	Graduate	42	18	Ministry of energy, Cameroon
6	Deputy-Manager	PhD	38	8	Solar Energy Technology, Cameroon
7	Deputy-Manager	Graduate	37	11	Instrumelec, Cameroon
8	Deputy-Director	PhD	52	25	ENEO, Cameroon
9	Assistant-Manager	Graduate	30	7	ENEO, Cameroon
10	Assistant-Manager	Graduate	35	11	SONATREL, Cameroon

**Fig. 5:**
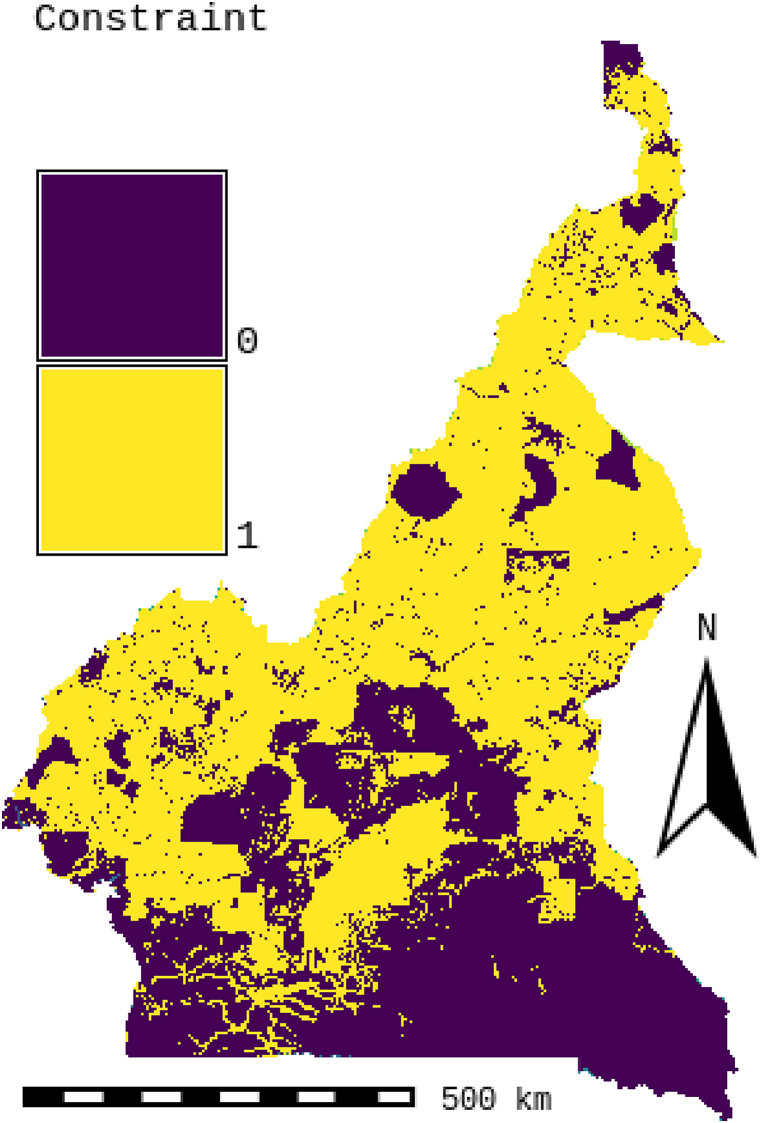
Constraint layer.

These maps were developed using information from Table 7, which corresponds to the factors and weightings selected to evaluate the solar energy production potential of CSP-grid-connect plants. The different weights corresponding to the different factors according to the different scenarios are presented in this same table. Using the AHP and the GIS, land suitability index maps are established to locate favorable sites for CSP-grid-connect solar plants in Cameroon. The results of the analysis are summarized in this part.

### 4.1 Determining suitable land

This study aimed to evaluate suitable sites for CSP-grid-connect solar plants in Cameroon using an AHP coupled GIS approach. The review of the literature and the expertise of the experts made it possible to select ten factors under several aspects, namely the climatic aspect (solar irradiation DNI, temperature); the orographic aspect (slope, elevation); the location aspect (proximity to electricity networks, roads, residential areas, and population density); and the watercourses aspect (proximity to watercourses and water lines) according to the requirements of the study for factories using CSP-grid-connect solar panels. The weights of the factors were obtained from the AHP approach based on the judgments of the ten experts collected during the interviews.

**Fig. 6:**
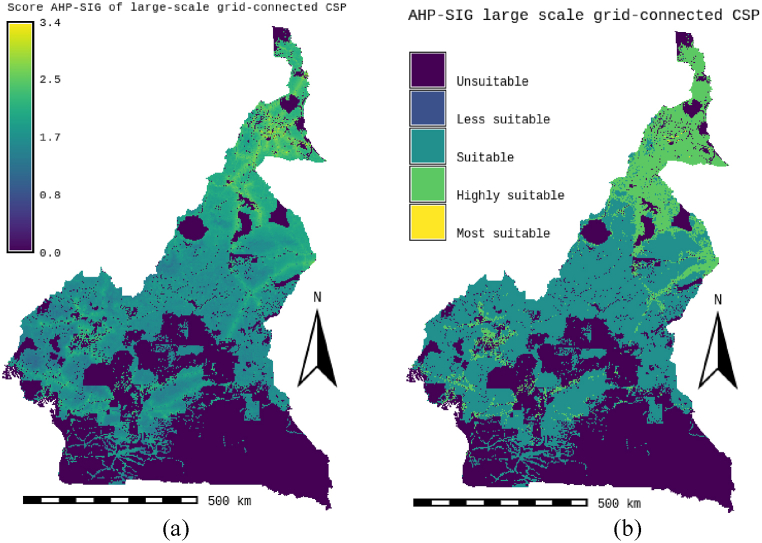
Score and Land suitability CPS-grid-connected (a) Score CSP-grid-connect (b) Land suitability CPS-grid-connect.

**Fig. 7:**
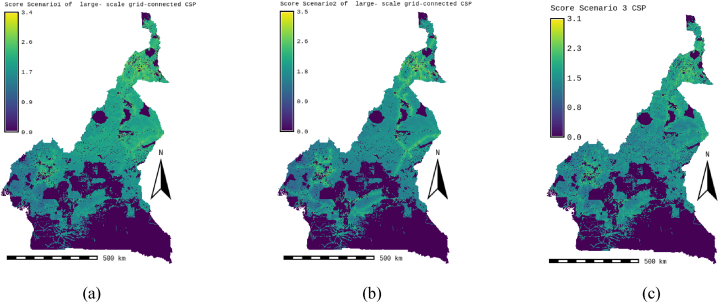
Score scenario 1,2 and 3; (a) Technic CPS-grid-connect scenario, (b) Socio-economic CPS-grid-connect scenario, (c) EQW CPS-grid-connect scenario.

**Fig. 8:**
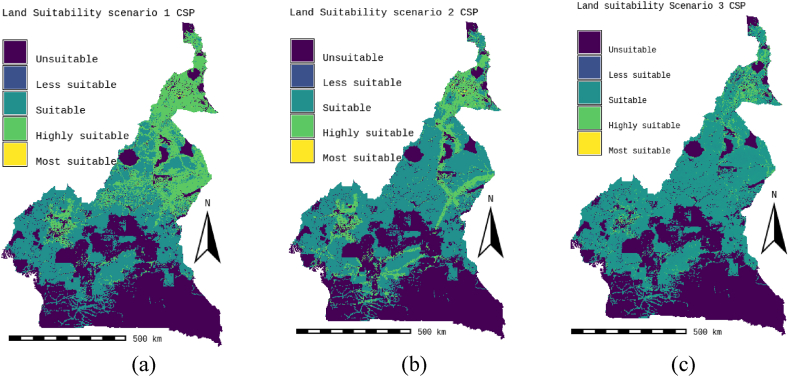
Land suitability Scenario 1,2 and 3; (a) Technic CPS-grid-connect scenario, (b) Socio-economic CPS-grid-connect scenario, (c) EQW CPS-grid-connect scenario.

**Fig. 9:**
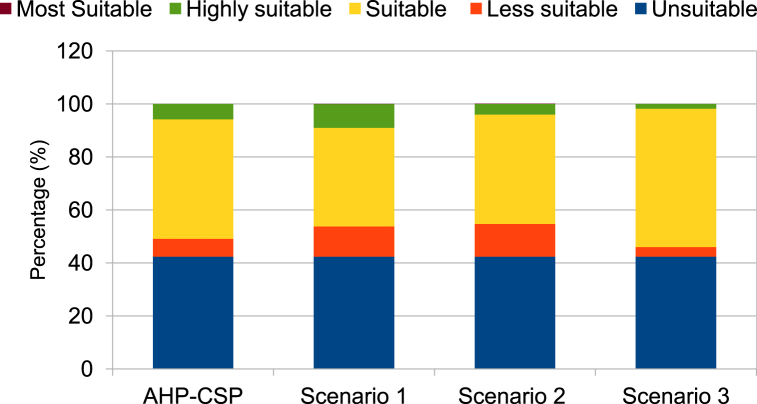
Graphical interpretation for land suitability areas for three sensitivity cases.

According to the results presented in Tables 7 and it is obvious that for the standard CSP-grid-connect scenario, the climatic aspect occupies a preponderant place (42 %). The rental aspect represents 28 %, followed by the water resource aspect representing 20 %, and finally the orographic aspect representing 10 %. It is also observed that the “solar irradiation DNI” factor is the most important, representing 22 % of the total. It is followed by the “temperature” factor by a weight of 20 %. The third most important factor is proximity to the electricity grid, with a weight of 14 %. The fourth factor is “proximity to watercourses and proximity to water lines”, each with a weight of 10 %. The factors “proximity to watercourses and proximity to waterlines” are fourth with weights of 10 % each. The factors “proximity to road network” and “proximity to residential areas” are fifth with weights of 10 % each. They are followed by the factors “elevation”, “population density” and “proximity to road networks” with respective weights of 7 %, 6 % and 5 %. It is possible to see that the elements “proximity to residential areas” and “slope” are the lowest in weight, representing 3 % each. All these characteristics can be found in Table 7. Fig. 6-a and 6-b present the scores and skill maps obtained using the GIS-AHP combination for the standard CSP-grid-connect scenario. Map in Fig. 6-a presents the final continuous aptitude map, and map in Fig. 6-b presents the discrete aptitude map, allowing the evaluation of the areas. After examining Table 8, Table 9, which list the statistical data and the theoretical solar potential for the various scenarios, it appeared that 43.35 % of land in Cameroon is considered “unsuitable” for the installation of CSP-grid-connect solar plants. After examining these same tables, it is also clear that 13.64 % of Cameroonian land is “very suitable” for CSP-grid-connect solar plants, with a CSP solar technical potential of approximately 9293.116 TWh/year. In contrast, 0.01 % are “the Most Suitable,” with a CSP solar potential of around 5800 TWh/year. There are 44.00 % and 0,001 % “Suitable” and “Less suitable,” respectively. This represents a total solar potential of approximately 37917.005 TWh/year for this standard CSP-grid-connect scenario. Fig. 7 shows the maps corresponding to the different scenarios. We recorded and reclassified these different cards to obtain discrete cards corresponding to the different aptitude indices mentioned above. Fig. 8 presents the recoded and reclassified cards for the different scenarios. Fig. 9 presents a graphical interpretation of land security zones and three sensitivity cases.

### 4.2 Sensitivity analysis

Three scenarios are taken into account in the sensitivity analysis: the technical scenario, which brings together climatic and orographic aspects (scenario 1), the socio-economic scenario, which corresponds to the aspect of location (scenario 2), and the equal weight scenario (scenario 3), which gives the same weight to all factors. Statistical data on land suitability zones and the theoretical CSP solar potential in TWh/year are listed in Table 8, Table 9

#### 4.2.1 Technical scenario (scenario 1)

According to the technical scenario, which combines the climatic and orographic aspects (Scenario 1), the climatic (59 %) and orographic (10 %) factors are the most important, while the location and watercourse aspects are the weakest (5 % and 26 %). Table 7 presents the weights of the factors linked to the technical scenario, while the ability maps resulting from the GIS-AHP combination are presented in Fig. 7, Fig. 8a. After examining Table 8, Table 9, which list statistical data and theoretical solar potential, it appeared that 43.35 % of land in Cameroon is considered “unsuitable” for the installation of CSP-grid-connect solar plants. This mainly concerns protected areas, land use, transport infrastructure, airports, waterways, orography, and the various restrictive criteria applied to the factors to respond to the socio-environmental, technical, and economic constraints that make it possible to optimize the choice of the site. According to these same Table 8, Table 9, it is also observed that 20.05 % of Cameroonian land is “highly suitable” for CSP-grid-connect solar plants, with a solar technical potential of approximately 13657.675 TWh/year. 01 % is “most suitable,” with a solar potential of approximately 8.123 TWh/year. The highest percentage, 37.58 %, corresponds to the “suitable” range, and only 0.001 % corresponds to the “less suitable " range. The solar potentials are, respectively, 24441.655 TWh/year and 3.147 TWh/year. This represents a total solar potential of approximately 38110.600 TWh per year for this socio-economic scenario. These results correspond perfectly to the technical scenario because the climatic factor (DNI solar potential and temperature) is the main element that impacts this scenario, with an overall weight of 69 %. By comparing the initial results of the study with the technical scenario, we see that the area of the zone corresponding to “highly suitable” increases from 78661 km^2^ to 115605 km^2^, which represents an increase of approximately 36944 km^2^. In addition, the area of the area corresponding to “suitable” decreases from 253773 km^2^ to 216738 km^2^, which represents a decrease of 37035 km^2^. In addition, the area of the “most suitable” zone increases from 48 km^2^ to 68 km^2^, which represents an increase of 20 km^2^. In addition, the surface area of the zone corresponding to “less suitable” increases from 0 km^2^ to 29 km^2^, an increase of approximately 29 km^2^. Moreover, solar irradiation (DNI) plays a vital role in this scenario. It is found that changing from the standard CSP-grid-connect scenario to the technical CSP-grid-connect scenario increases “highly suitable,” “most suitable,” and “less suitable” and a reduction in the “suitable” zone.

#### 4.2.2 Socio-economic scenario (scenario 2)

Location factors, such as proximity to electricity grids, roads, residential areas, and population density, are most important (57 %) in the socio-economic scenario corresponding to the location aspect (Scenario 2), while climatic, orographic, and watercourse factors are the lowest (26 %, 5 %, and 12 %). Table 7 presents the weights of the various factors linked to the socio-economic scenario, while the aptitude maps obtained from the GIS-AHP combination are presented in Fig. 7-b and 8-b. After examining Table 8, Table 9, which list the statistical data and the theoretical CSP solar potential, respectively, it is evident that 43.35 % of land in Cameroon is deemed “unsuitable” for the installation of CSP-grid-connect plants. The analysis of these same tables also reveals that 12.35 % of Cameroonian land is “highly suitable” for CSP-grid-connect solar plants, with a solar technical potential of 8417.215 TWh/year. In addition, 0.03 % are “most suitable,” with a solar potential of approximately 21,414 TWh/year. Finally, respectively, 45.22 % and 0.04 % are “suitable” and “less suitable,” with solar potentials of 29,409.661 TWh/year and 26.935 TWh/year, respectively. This represents a total solar potential of approximately 37,875.225 TWh/year in this socio-economic context. Comparing the results of the standard scenario with the socio-economic scenario, we see that the surface area of the zone corresponding to “highly suitable” decreases. from 78,661 km^2^ to 71,247 km^2^, a decrease of approximately 7414 km^2^; the area of the zone corresponding to “suitable” increases from 253773 km^2^ to 260793 km^2^, an increase of 7020 km^2^; the area of the zone corresponding to “most suitable” increases from 48 km^2^ to 176 km^2^, an increase of 128 km^2^; the area of the zone corresponding to “less suitable” increases from 0 km^2^ to 247 km^2^, an increase of approximately 247 km^2^. Moreover, proximity to the electricity grid plays an essential role in this scenario. To avoid losses associated with the transmission of electrical energy over long distances, it is very cost-effective to build CSP solar plants close to electrical transmission lines to avoid the construction of new lines to transport the electricity. This will improve transport and reduce investment expenses. In summary, it is possible to conclude that the transition from the initial scenario to the socio-economic scenario leads to a reduction in the “highly suitable” zone and an increase in the “most suitable,” “suitable,” and “less suitable” zones.

#### 4.2.3 Equal weight scenario (scenario 3)

In the equal weight scenario (Scenario 3), all factors receive the same weight, i.e., 10 % for each of the 10 factors. According to this scenario, climatic factors represent 20 %, orographic factors 20 %, location factors 40 %, and watercourse factors 20 %. Table 7 presents the weights of the factors corresponding to the equal-weight scenario, while the ability maps resulting from the GIS-AHP combination are presented in Fig. 7-c and 8-c. After Having examined Table 8, Table 9, which list the statistical data and theoretical solar potential, respectively, it is evident that 43.94 % of land in Cameroon is deemed “unsuitable” for the installation of large-scale CSP solar power plants. It is also observed that 12.35 % of Cameroonian land is “highly suitable” for large-scale CSP solar farms, with a solar technical potential of approximately 2685.276 TWh/year. However, 0 % are “most suitable,” with a solar potential of approximately 0 TWh/year. On the other hand, 53.55 % and 0.16 % are respectively “suitable” and “less suitable,” with solar potentials of 34828.734 TWh/year and 909.23 TWh/year. This represents a total solar potential of approximately 37613.289 TWh/year for this equal-weight scenario. Comparing the result of the standard CSP-grid-connect scenario to the equal-weight scenario, it appears that the area of the zone corresponding to “highly suitable” decreases from 78,661 km^2^ to 22,729 km^2^, a decrease of approximately 55,932 km^2^; the surface area of the zone corresponding to “suitable” increases from 253,773 km^2^ to 308,848 km^2^, an increase of 55,075 km^2^; the surface area of the zone corresponding to “most suitable” decreases from 48 km^2^ to 0 km^2^, i.e. a reduction of 48 km^2^; the surface area of the zone corresponding to less “suitable” increases from 0 km^2^ to 909 km^2^, an increase of approximately 909 km^2^. We note that this equal-weight scenario corresponds to 40 % of the technical factors (climatic and orographic), 40 % of the socio-economic aspect (location), and 20 % of the watercourses. In short, we can note that the transition from the standard CSP-grid-connect scenario to the equal weight scenario leads to a reduction in the “highly suitable” and “most suitable” zones and an increase in the “suitable zones” and “less suitable”.

**Fig. 10:**
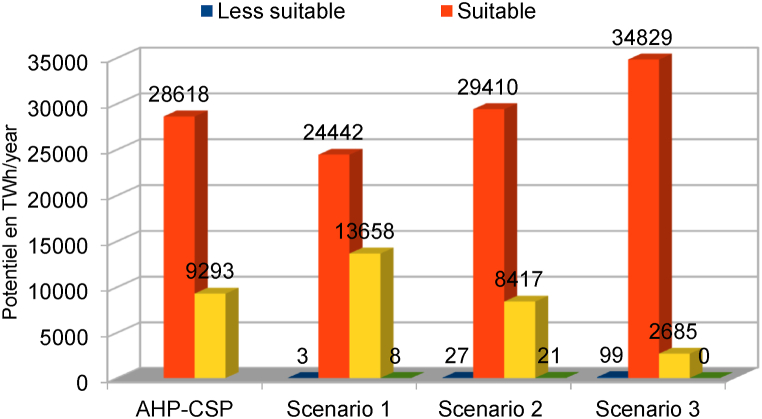
Theoretical potential of solar energy in Cameroon based on site suitability indices and sensitive analysis.

In summary, by examining the various situations with the standard CSP-grid-connect scenario, it is observed that the technical potential of CSP solar increases by approximately 193.595 TWh per year when moving from the standard scenario (37917.005 TWh/year) to the technical scenario (38110.600 TWh/year). Compared to the standard scenario, this potential decreases by 41.78 TWh/year and by 303.716 TWh/year when we move from the socio-economic scenario (37875.225 TWh/year) to the equal-weight scenario (37613.289 TWh/year). Fig. 10 shows the theoretical potential of solar energy in Cameroon based on on-site security indicators and in-depth analysis. The results can guide investors to make decisions based on their criteria.

Concentrated solar power stands out as the most promising technology for generating substantial amounts of heat at medium to high temperatures. Concentrated Solar Power (CSP) holds significant potential in renewable energy, utilizing mirrors or lenses to focus a vast expanse of sunlight, or solar thermal energy, onto a compact area [82]. The concentrated heat serves as a heat source for a conventional power plant. A notable benefit of this technology is its ability to generate substantial amounts of heat at medium to high temperatures, which is perfect for industrial uses. Moreover, Concentrated Solar Power (CSP) can store the generated heat, enabling electricity production even in the absence of sunlight. This attribute renders CSP a dependable and adaptable renewable energy source. It is crucial to acknowledge that the potential of Concentrated Solar Power (CSP) is significant, yet its deployment and efficacy are influenced by various elements. These include the geographical location, which is optimal in regions with abundant direct sunlight, the initial costs of investment, and environmental impacts. Scenario testing that considers environmental factors and other parameters could offer a broad array of decision-making options, depending on the goals investors aim to accomplish. Indeed, the detrimental impacts of climate change should concern everyone, as contributing to the mitigation of climate change's effects, particularly on communities, is crucial [82].

### 4.3 Regional analysis

**Fig. 11:**
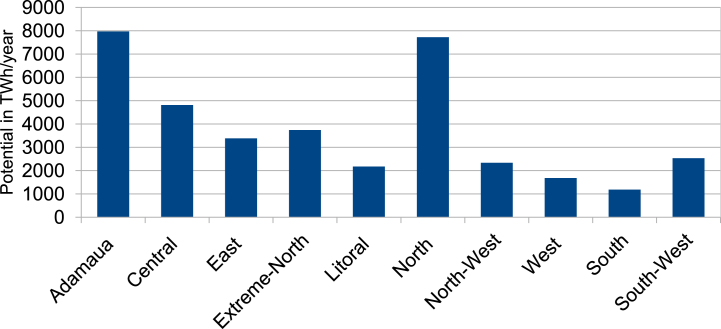
Power generation potential solar CSP in regions on Cameroon.

**Fig. 12:**
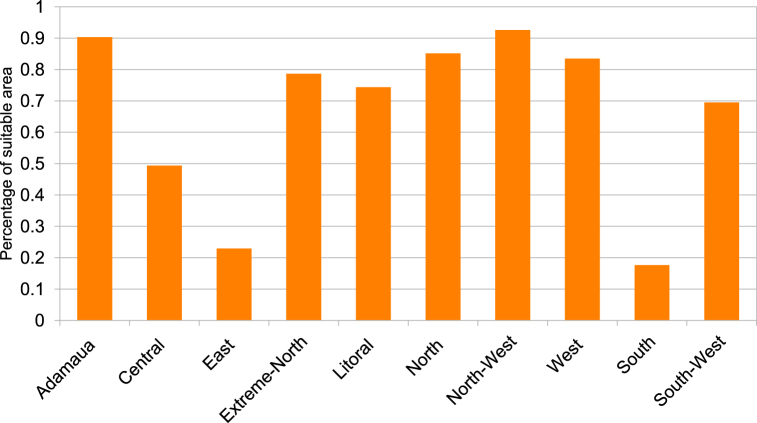
Percentage of suitable area of solar CSP for ten regions of Cameroon.

By examining in detail Table 10, which presents the statistical results and the theoretical power potential for the CSP-grid-connect systems, it is observed that the highest theoretical power potentials are observed, respectively, in Adamawa and the north of Cameroon, with respective values of 7969.273 TWh/year and 7723.886 TWh/year for suitable total areas of approximately 71088 km^2^ and 682019 km^2^. Indeed, these two regions receive the largest DNI irradiation in the country. It is evident in this same table that the lowest theoretical power potentials are observed, respectively, in the South and the West, with values of approximately 1184.856 TWh/year and 1679.346 TWh/year for suitable total areas of approximately 10552 km^2^ and 14943 km^2^. Fig. 11, Fig. 12 illustrate the theoretical CSP solar technical possibilities by region and the percentage of surface area suitable for CSP-grid-connect sites in Cameroon. Theoretical power potential is linked to surface area. The West region has the largest area of Cameroon, with an area of 17904 km^2^, which results in low theoretical power in this region. When assessing the percentage of suitable area compared to the total area per region, it is observed that the percentages lower are mainly observed in the South and the East, with respective values of 17.63 % and 22.94 %, as well as a theoretical power potential per square kilometer of 0.020 TWh/year/km^2^ and 0.026 TWh/year/km^2^. This is explained by the limitations of the study. Indeed, these two zones are essentially forested, which constitutes a limiting criterion for the preservation of the environment. The highest rates are observed in the North-West and Adamawa, with respectively 92.60 % and 90.33 %, as well as a theoretical power potential per square kilometer of 0.104 TWh/year/km^2^ and 0.101 TWh/year/km^2^. Sunshine in these two regions is very high, and the electricity and road network infrastructure is extensive. This may explain the higher percentages compared to other areas. Overall, the in-depth analysis of Table 10 reveals that, apart from the South and East regions, more than 49 % of the land in each region is suitable for the construction of CSP-grid-connect plants in Cameroon. The varied percentages and power generation potential solar CSP in ten regions of Cameroon are shown in Fig. 11, Fig. 12. In the CSP-grid-connect solar system, the regions having suitability of “Less Suitable,” “Suitable,” “Highly Suitable,” and “Most Suitable” have a total theoretical technical potential of approximately 4242.25 TWh/year, 29328.658 Twh/year, 3965.25 TWh/year, and 5793 TWh/year, with respective areas of 38852 km^2^, 260075 km^2^, and 33564 km^2^.

**Fig. 13:**
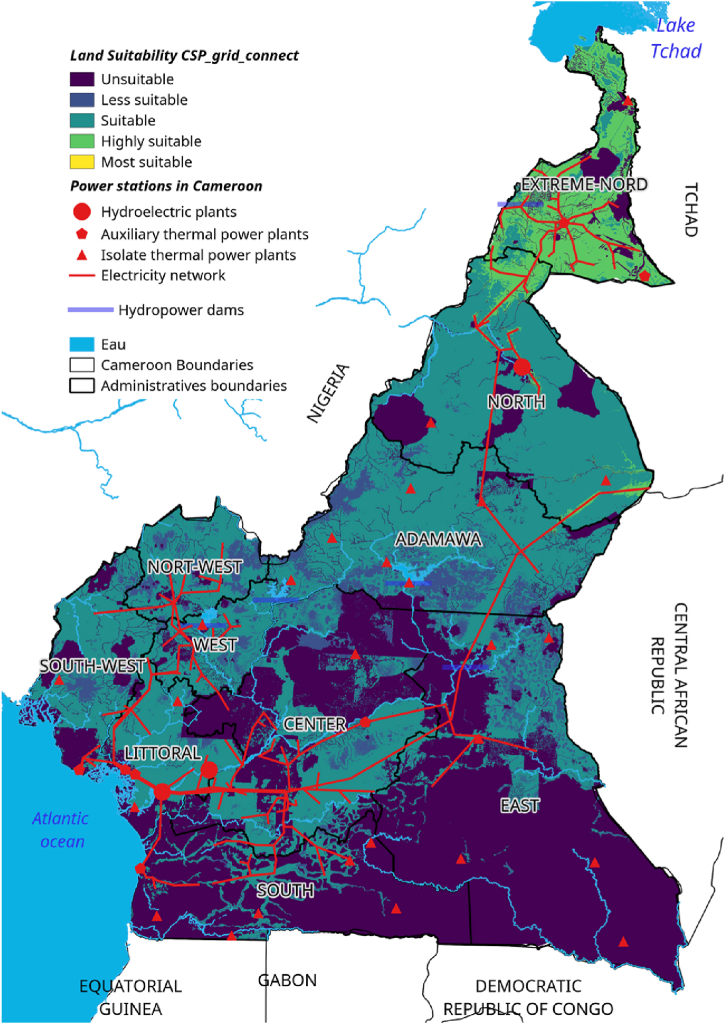
Land suitability CSP-grid-connect and power stations in Cameroon.

Superposition of existing power plants on the adaptability map shows that isolated or additional solar thermal power plants are mainly in “Suitable” zones in the 9 regions of Cameroon. This is illustrate by Fig. 13. In the Far North region of Cameroon, these thermal power plants are mainly located in the “Highly Suitable” zone. Although the Far North region of Cameroon has the highest CSP solar potential, there is a huge gap between supply and demand. This deficit leads to power cuts that can last several weeks and poor power quality. The construction of CSP solar power plants could therefore be a solution to the problems of access to electricity encountered by the populations of Cameroon. However, the solar CSP potential is not very high, will a solar PV-CSP or wind-CSP hybridization or even PV-CSP-Wind be able to improve the profitability of a site in Cameroon? An exploration of this issue will be made in future work.

## 5 Conclusion

In this study, an approach using the geographic information system (GIS) and multi-criteria analysis (MCDM) is presented to map sites favorable to the installation of CSP-grid-connect solar power plants in Cameroon. Despite the importance of DNI solar irradiation and temperature in the decision to integrate large-scale CSP solar farms, other elements, such as proximity to the electricity grid and proximity to waterways, favor the optimal layout of CSP sites. We studied the most suitable areas for the installation of CSP in Cameroon using the MCDM-AHP approach and the GIS GRASS GIS 7 software. According to the results, it was found that nearly 42.35 % of the total area of the country was unsuitable for the installation of CSP solar plants connected to the existing electricity network. We evaluate the sites favorable for the installation of CSP plants connected to the network according to their compatibility with the deployment of solar systems, taking into account orographic, climatic, topographical, social, and environmental constraints and factors likely to hinder or facilitate the development of CSP solar production. According to this study, large-scale CSP connected to the grid in favorable areas has a potential total technical capacity of approximately 29765.176 TWh per year. The major results of this research are as follows.

• The use of the AHP to evaluate the various weights of the standard AHP-CSP scenario reveals that the climatic criterion plays a preponderant role in the choice of CSP-grid-connect plants, with a percentage of 42 %. The orographic criterion is the one that has the least impact, with a percentage of 10 %. In Cameroon, 42.35 % of the area is considered “unsuitable,” 0.00 % as “less suitable,” 44 % as “suitable,” 13.64 % as “very suitable,” and only 0.01 % as “most suitable.” The standard scenario presents a technical potential for CSP solar energy production of 37917.005 TWh per year.
• The technical scenario (Scenario 1) presents an increase in the technical potential for solar energy production compared to the standard AHP-CSP scenario, according to the sensitivity analysis based on the variation of the weights. Economic and balanced situations lead to a decrease in this potential. In addition, the ranges corresponding to “less suitable” and “more suitable” present the lowest potentials in all scenarios.

Despite the efforts made to make geospatial data available for our study, there are inherent uncertainties due to the nature of global spatial data and, in particular, the updating of spatial data in Cameroon. These uncertainties are associated with the whole MCDM decision-making process. The objective is to define the limits, calculate the values of the spatial criteria, and choose the appropriate weights. In this study, we only evaluated the uncertainty and sensitivity of a primary source depending on the weight of the criteria. This study will be able to provide support for decision-making in the country's energy policy, in particular by persuading and strengthening the confidence of investors in the establishment of CSP solar plants. This study could provide support for decision-making in the country's energy policy, in particular by persuading and strengthening the confidence of investors in the establishment of CSP solar plants. Given the obsolescence of data on the occupation of the ground in Cameroon, it will be essential to update this data to obtain results closer to reality on the ground. Nevertheless, it should be emphasized that the validation of the results of this study, which identify sites favorable to the installation of CSP solar plants in Cameroon, is carried out after a site visit.

Considering that Cameroon has superior GHI (Global Horizontal Irradiance), irradiation compared to DNI, hybridizing PV with CSP solar technologies could optimize site profitability. Introducing additional parameters, such as the “distance to existing power plants,” may further refine site selection for CSP. Moreover, a methodology that employs a fuzzy approach in conjunction with the Monte Carlo method to identify optimal sites for hybrid renewable energy power plants is nearing completion.

## Declaration of competing interest

The authors declare that they have no known competing financial interests or personal relationships that could have appeared to influence the work reported in this paper.
